# Comparison of the six-minute walk test performed over a 15 and 30 m course by children with cerebral palsy

**DOI:** 10.1186/s12891-022-05944-z

**Published:** 2023-01-17

**Authors:** Joanna Krasny, Marek Jozwiak, Elisabet Rodby-Bousquet

**Affiliations:** 1 Department of Pediatric Orthopedics and Traumatology, Poznan University of Medical Sciences, Poznań, Poland; 2 Department of Clinical Sciences Lund, Orthopaedics, Lund University, Lund, Sweden; 3 Centre for Clinical Research, Uppsala University-Region Västmanland, Västerås, Sweden

**Keywords:** Cerebral palsy, Children, Adolescents, Walking, Measurement

## Abstract

**Background:**

The aim of this study was to compare performance on the six-minute walk test (6MWT) performed over 15 m and 30 m courses by children and youths with cerebral palsy (CP).

**Methods:**

Children and youths with CP at Gross Motor Function Classification System levels I–IV performed the 6MWT in a straight 15 m-long corridor (first trial) and 30 m-long corridor (second trial). The intraclass correlation coefficient (ICC) and Bland-Altman plots were used to evaluate the agreement between the 6MWT results for the two corridor lengths.

**Results:**

We included 82 children and youths with CP (36 girls, 46 boys), with a mean age of 11.7 years (SD 4.2, range 5–22 years). There was high agreement between the results of the two 6MWTs: ICC 0.93 (95% confidence interval 0.76–0.97). The total walking distance was longer for the 30 m course (median 399 m, range 44–687 m) than the 15 m course (median 357 m, range 24–583 m).

**Conclusions:**

We observed good agreement for the performance of the 6MWT in the 15 m and 30 m courses, although the total walking distance was greater for the 30 m course. We recommend that the same distance is used when evaluating changes in walking ability for an individual child. Both distances are appropriate when measuring endurance in children and youths with CP.

## Background

Cerebral palsy (CP) is caused by a nonprogressive brain injury early in life and affects primarily movement and posture but can also cause activity limitations [1]. Children with CP are usually born without deformities, but secondary musculoskeletal complications tend to develop in childhood and increase in severity with time. CP is associated with musculoskeletal and neurological disorders that affect a child’s motor function, walking ability, and gait pattern [2], which can also impair endurance [3]. Children with CP are less physically active than their peers [4–6]. According to the International Classification of Functioning (ICF), walking disabilities and reduced physical activity can limit participation by children with disabilities [7–11].

Field walking tests are commonly used to evaluate exercise capacity in clinical practice [12]. These tests are simple and easy to perform, and do not require any special equipment. The six-minute walk test (6MWT) is a submaximal exercise test used to assess aerobic capacity and endurance, and to indicate an individual’s functional capacity for daily physical activity [13]. Holland et al. compiled a standard operating procedure for the 6MWT in patients with chronic respiratory diseases. The test should be performed along a flat, straight course with a hard surface measuring at least 30 m in length [12]. However, it can be challenging to find a suitable 30 m stretch in an indoor clinical setting. The recommendations given by the American Thoracic Society (ATS) as guidelines for the 6MWT were not intended to limit the use of alternative protocols for research studies [13]. Thus, the literature contains several modifications of the 6MWT in terms of conditions (indoor or outdoor [14]), distance (20–50 m [15–17]), and patients’ medical conditions (cardiac, pulmonary, or neurological disorders [18–20]).

The 6MWT is a reliable test for children with CP [21]. Studies show that age and gross motor function correlate with 6MWT performance in children with CP [22, 23]. Maher et al. [24] evaluated the reliability of the 6MWT in young ambulant people with CP. However, their results were based on a test performed on a 10 m course. Differences in the course distance can lead to variability and affect the reliability of the measurements. The aim of this study was to compare 6MWT performance when performed by children and youths with CP over 15 m and 30 m courses.

## Methods

The 6MWT was performed by children and adolescents with CP at Gross Motor Function Classification System (GMFCS) levels I–IV. They were recruited at the Rehabilitation Centre, Department of Pediatric Orthopedics and Traumatology, Poznan University of Medical Sciences, Poland, between 25 November 2019 and 21 January 2020.

The inclusion criteria were a diagnosis of CP, the ability to walk independently with or without a device for 6 min (no assistance was required with turning or steering), and the ability to understand and follow instructions. The exclusion criteria were the inability to walk or acute pain that could affect ambulation at the time of the examination.

For all patients, the 15 m 6MWT trial was performed on the first day of a 2-week rehabilitation training program, and the 30 m distance trial was performed at the same time of day on the second day. Both tests were performed before any rehabilitation training began. All examinations and testing were administered by six physiotherapists, who supervise the 6MWT regularly, and using an established protocol.

The 6MWT was performed indoors, on a flat, straight, hard-surfaced 15 m-long corridor (first trial) and a 30 m-long corridor (second trial). The length of the walking course was lined by tape every 3 m. The turnaround points were indicated by orange cones at both ends of the course. The children and adolescents wore appropriate comfortable shoes for walking and used their orthoses or usual walking aids (cane, walker, etc.) during both the first and second trials. Before each test, the participant sat in a chair and relaxed for 10 min. No warm-up was performed.

Before the test, the examiner instructed the participants about completing the test, including the statement, “You should walk as fast as you can, but not run.” Each participant was then asked to walk the course between the cones for 6 min. The examiner cautioned the participants not to run but encouraged them with standardized phrases such as, “You are doing a great job!” and “Keep going!”. During the trial, the participants were permitted to stop or slow down and to resume walking as soon as possible, but the timer was not stopped. The final length of the trial was calculated by counting the number of laps and calculating the measured distance from the starting position to the stopping point in meters.

The study was approved by the ‘Ethical Committee’ of Poznan University of Medical Sciences (nr 244/20). All the methods were performed under relevant guidelines and regulations or under Declaration of Helsinki. Parents or legal guardians of each patient enrolled into the study signed the written consent form.

### Statistical analysis

The intraclass correlation coefficient (ICC) [25] with the two-way random and absolute agreement definition was used to evaluate the agreement between the 6MWT performance for the 15 and 30 m courses. The mean with standard deviation (SD), median with range, standard error of the differences with 95% confidence intervals (CI) were used to analyze systematic differences between the two distances. Bland-Altman plots [26] were used to estimate the difference between the two distances against their mean, with limits of agreement based on ±2 SD. IBM SPSS Statistics (version 26.0) was used for all statistical analyses.

## Results

We included 82 children and youths with CP classified at GMFCS levels I–IV; 36 were girls and 46 were boys. Their age range was 5–22 years (mean age 11.7 years [SD 4.2]) (Table 1).

**Table 1:** Characteristics of the participants

	***N***	%
Age (years), mean [SD]	11.7	[4.2]
Girls	36	43.9
Boys	46	56.1
GMFCS I	18	22
GMFCS II	36	43.9
GMFCS III	19	23.2
GMFCS IV	9	11
Total	82	100

*GMFCS* Gross Motor Function Classification System, *SD* Standard deviation

There was a high agreement between the results of the 6MWT over the 15 and 30 m courses: ICC 0.93 (95% CI 0.76–0.97). The median total distances were 399 m (range 44–687 m) for the 30 m course and 357 m (range 24–583 m) for the 15 m course (Table 2). The same was observed within all GMFCS levels; that is, the median distances walked were longer when using the 30 m than the 15 m corridor (Table 3).

**Table 2:** Total walking distance (m) in the 6MWT over the 30 m and 15 m courses

	6MWT	
	30 m	15 m
Mean	365.1	330.2
SD	158.5	142
Median	399	357
Percentiles		
25	300	251.3
75	459	445.3
Minimum	44	24
Maximum	687	583

*6MWT* Six-minute walk test, *SD* Standard deviation

**Table 3:** Total walking distance (m) in the 6MWT by children at GMFCS levels I–IV over the 30 m and 15 m courses

			6MWT	
			30 m	15 m
GMFCS I	Median		518	465
(*n* = 18)	Percentiles	25	454	433
		75	587	521
	Minimum		420	328
	Maximum		687	583
GMFCS II	Median		414	386
(*n* = 36)	Percentiles	25	350	324
		75	456	437
	Minimum		74	72
	Maximum		630	495
GMFCS III	Median		304	279
(*n* = 19)	Percentiles	25	120	135
		75	360	306
	Minimum		44	45
	Maximum		417	363
GMFCS IV	Median		138	105
(*n* = 9)	Percentiles	25	65	66
		75	171	190
	Minimum		55	24
	Maximum		287	306

*6MWT*, Six-minute walk test; *GMFCS*, Gross Motor Function Classification System

**Fig. 1:**
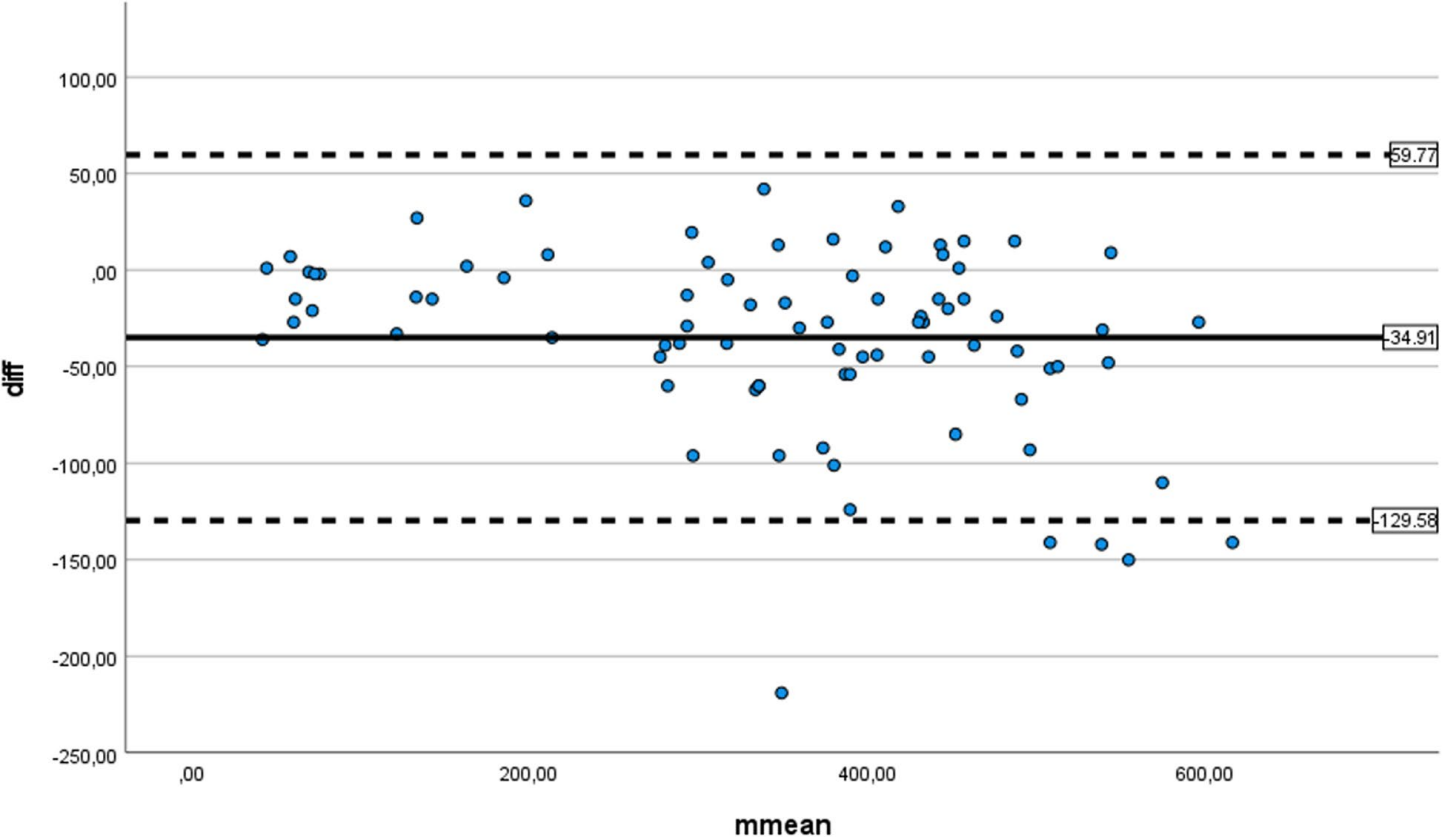
Bland-Altman plots of differences against mean with limits of agreement of ±2 standard deviations

The mean difference between 15 and 30 m distances was − 34.91 (95% CI −45.31 to −24.51), SD 47.34 m, and standard error 5.23. Bland-Altman plots of differences against mean ± 2 SD for the two methods indicate that some children with the lowest average distances performed better at the 15-m distance, while the opposite was seen for most children with higher average distances (Fig. 1).

## Discussion

We found a high level of agreement in 6MWT performance between the 15 m and 30 m walking courses in children and youths with CP. Even though most children preformed slightly better at the 30 m course, some children with lower scores on the 6MWT (typically GMFCS IV) seemed to perform slightly better at the 15 m course. The participants in this study represented different ages and GMFCS levels (I–IV). All participants were ambulant and were allowed to use their usual walking aids, orthotics, and shoes during the tests. Previous studies have reported on the utility of the 6MWT in people with CP. Fitzgerald et al. [27] described a reference range of values in ambulant children with spastic CP at GMFCS levels I–III and their healthy peers. The main modification from the original ATS protocol was a 70 m walking trail. We found a slightly different distribution of values across the GMFCS levels than those reported by Fitzgerald et al. Fiss et al. [22] reported on the developmental trajectories and reference percentiles for the 6MWT in 3–12-year-old children with CP at GMFCS levels I–III. Even though the participants in our study were older, the results were consistent with the developmental trajectories noted by Fiss et al. [22].

We could not find any studies evaluating agreement between the 6MWT at two different distances for children with CP. However, a previous study by Sciurba et al. [28] involved 761 participants who performed the 6MWT at 17 clinical centers after lung volume reduction surgery. The performance on the test varied according to the dimensions of the walking trail. The authors concluded that patients achieved a longer distance on the longer courses. Our finding is consistent with this result. However, Sciurba et al. [28] noted that it seems less important to standardize the length of the course provided it exceeds the minimum of 50 ft, which is around 15 m.

In our study, 70% of participants improved their walking distance on the second day, which may reflect a learning effect, as reported by Jay et al. [29] and Trooster et al. [17]. Jay et al. [29] analyzed data collected from a population-based study involving 3805 individuals using a 100-ft course and observed a mean 15% improvement when the test was performed on two successive days. However, they noted that this effect is not important when determining cross-sectional correlations or when using the results as a baseline predictor of later events. Trooster et al. [17] reported a similar learning effect in a study of 51 healthy subjects who performed the 6MWT twice on a 50 m course with 2.5 h between the two tests. The distance covered on the second test was on average 8% greater than on the first test.

The protocol of the 6MWT should be standardized in terms of encouragement. The positive effect of encouragement in the 6MWT was reported by Guyatt et al. [30], who found that encouragement given every 30 s during the walking test was associated with a significant increase in the distance walked. Jay et al. [29] concluded that a longer distance may be expected when non-standardized encouragement is given. To reduce potential effects of non-standardized encouragement, we used a standardized protocol where encouragement was given every 10 seconds to all children on both occasions.

One limitation of this study was that the 30 m test was performed the day after the 15 m test in all participants. The systematically longer distance covered in the 30 m test may reflect the effects of a longer course or a learning effect, or both. However, the aim of this study was to determine the agreement between tests over these two distances, which was shown to be high. Another limitation of this study was the use of an indoor space that was unfamiliar to the participants. An unfamiliar setting can influence children, especially those with disabilities. Patients walking in a gait laboratory may have a different gait pattern from that observed by parents or caregivers at home. Therefore, assessment of walking distance should preferably be performed in a natural environment using a portable device [31].

## Conclusions

Walking tests are a part of the assessment of children with CP. It is important to consider the results of assessments such as the clinical examination and gait analysis when making decisions about surgical treatment and rehabilitation. This study showed a high agreement between the 6MWT performed on 15 m and 30 m courses in children and youths with CP. The total walking distance may be slightly shorter when the test is performed over a 15 m course. Therefore, we recommend that the same distance is used when evaluating potential changes for an individual child. Our findings suggest that both tests are appropriate and reliable methods when measuring endurance in children and youths with CP.

## Data Availability

The data that support the findings of this study are available from the corresponding author upon reasonable request.

## Abbreviations

CP: Cerebral palsy
6MWT: Six-minute walk test
ATS: American Thoracic Society
GMFCS: Gross Motor Function Classification System
ICC: Intraclass correlation coefficient
SD: Standard deviation
